# High expression of intratumoral stromal proteins is associated with chemotherapy resistance in breast cancer

**DOI:** 10.18632/oncotarget.10894

**Published:** 2016-07-28

**Authors:** Tingting Wang, Supriya Srivastava, Mikael Hartman, Shaik Ahmad Buhari, Ching-Wan Chan, Philip Iau, Lay Wai Khin, Andrea Wong, Sing-Huang Tan, Boon-Cher Goh, Soo-Chin Lee

**Affiliations:** ^1^ Cancer Science Institute, National University of Singapore, Singapore; ^2^ Department of Haematology and Oncology, National University Cancer Institute, National University Health System, Singapore; ^3^ Department of Surgery, National University Cancer Institute, National University Health System, Singapore; ^4^ Saw Swee Hock School of Public Health, National University of Singapore, Singapore

**Keywords:** breast cancer, chemotherapy resistance, cancer-associated stromal protein, integrin β, mTOR pathway

## Abstract

We studied the changes of intratumoral stromal proteins including THBS1, TNC, FN, SPARC and α-SMA, following neoadjuvant chemotherapy. The underlying mechanisms by which THBS1 and TNC regulated resistance to docetaxel were further studied using functional studies. 100 patients with newly diagnosed breast cancer were treated with alternating sequential doxorubicin and docetaxel. Immunohistochemistry (IHC) staining for stromal proteins was performed on pre- and post-treatment core biopsies respectively. THBS1 and TNC were further validated with IHC in an independent cohort of 31 patients. A high baseline combined expression score of the 5 stromal proteins predicted independently for poor progression-free (HR_adjusted_ 2.22, 95% CI 1.06–4.64) and overall survival (HR_adjusted_ 5.94, 95% CI 2.25–15.71). After 1–2 cycles of chemotherapy, increased expression of THBS1, TNC, FN, SPARC and α-SMA was seen in patients with subsequent pathological lymph node involvement at surgery. Increased expression of THBS1 and TNC compared to baseline was also seen in intrinsically resistant tumors, but not in sensitive ones. Both THBS1 and TNC-associated chemoresistance were confirmed in an independent validation cohort. Exogenous THBS1 and TNC protected MCF-7 cells against proliferation inhibition induced by docetaxel through activating integrin β1/mTOR pathway. Thus, up-regulation of THBS1, TNC, FN, SPARC and α-SMA following neoadjuvant chemotherapy was associated with chemotherapy resistance in breast cancer patients. Functional studies showed THBS1 and TNC to mediate chemoresistance through the integrin β1/mTOR pathway, suggesting that therapies targeting integrin β1/mTOR pathway may be a promising strategy to overcome chemotherapy resistance.

## INTRODUCTION

Chemotherapy is a cornerstone treatment in patients with early and advanced breast cancer. However, primary and acquired resistance to chemotherapy exists. Increasing evidence indicates that cancer cells are not the only determinants for tumor growth; intratumoral stroma also plays an important role in tumor progression and chemotherapy response [1–3], especially in tumors with intense desmoplastic reaction. For example, enrichment in stroma-related gene pathways in pancreatic ductal adenocarcinoma was associated with poor survival and resistance to gemcitabine [2]. Similarly, stromal gene signatures may predict resistance to anthracyclines in breast cancer [3]. Breast cancer has also been classified according to their stromal gene profile, which provides additional prognostic information independent of conventional tumor features, such as estrogen receptor (ER) or human epithelial growth factor 2 (Her2/neu) status [4].

Intratumoral stroma directly stimulates tumor cell proliferation by secreting various growth factors, hormones and cytokines, and mainly contributes to the invasiveness, metastasis as well as treatment response by inducing epithelial-mesenchymal transition, a known epigenetic program leading cells to manifest a motile and proteolytic phenotype [5]. The main proteins expressed by intratumoral stroma comprise thrombospondin 1 (THBS1), tenascin C (TNC), fibronectin (FN), secreted protein acidic and rich in cysteine (SPARC) and smooth muscle actin-α (α-SMA), which have been reported to be associated with increased invasiveness [6–8], treatment response [9, 10] and poor prognosis [11–13] in breast cancer.

The aim of this study was to determine the changes in a panel of stromal proteins within the tumor, including THBS1, TNC, FN, SPARC and α-SMA, following neoadjuvant chemotherapy in newly diagnosed breast cancer patients using immunohistochemistry (IHC) staining. Stromal proteins expression in both baseline and post-treatment tumors were correlated with survival; stromal proteins expression at baseline and their changes from baseline were correlated with ER status, intrinsic chemotherapy response, and pathological lymph node (PLN) metastasis at surgery, respectively. Among these 5 stromal proteins, THBS1 and TNC were identified to be more relevant to chemoresistance. As such, chemotherapy-induced changes in THBS1 and TNC expression by IHC were further validated in another independent clinical dataset. These two proteins were also studied further in MCF-7 breast cancer cell line to explore the underlying mechanisms by which they regulate chemotherapy resistance.

## RESULTS

### Clinico-pathological characteristics (Table 1)

Female patients with newly diagnosed locally advanced or metastatic breast cancer were recruited into two separate prospective phase II studies: a primary and an independent validation cohort. In the primary cohort, 100 patients were randomized to one of two alternating sequences of doxorubicin (A) and docetaxel (T) every three weeks for six cycles, followed by breast cancer surgery. Pre-, post-cycle-1- and post-cycle-2- chemotherapy tumor core biopsies were obtained. In the validation cohort, 31 patients were treated with four cycles of neoadjuvant docetaxel administered 3-weekly. Pre-, post-cycle-1- and post-cycle-4- chemotherapy tumor core biopsies were collected. The median age of patients in both the primary and validation clinical cohorts was 50 years (range 26-68 and 31-63 respectively). Mean progression free survival (PFS) and overall survival (OS) were 45.3 [95% CI 38.4–52.3] and 59.4 [95% CI 53.1–65.7] months for the primary cohort, and 48.0 [95% CI 38.7–57.3] and 52.8 [95% CI 44.7–60.8] months for the validation cohort, respectively.

**Table 1:** Clinicopathological characteristics of the primary cohort (*n* = 100) and validation cohort (*n* = 31)

	Primary cohort (*n*, %)	Validation cohort (*n*, %)
**Age**		
< 50	53 (53)	15 (48)
> = 50	47 (47)	16 (52)
**Ethnicity**		
Chinese	65 (65)	14 (45)
Malay & others	35 (35)	17 (55)
**Tumour grade**		
1	10 (10)	2 (7)
2	49 (49)	10 (32)
3	41 (41)	19 (61)
**T4 stage**		
No	26 (26)	19 (61)
Yes	74 (74)	12 (39)
**Metastasis**		
No	69 (69)	22 (71)
Yes	31 (31)	9 (29)
**Treatment arm**		
A-T-A-T-A-T	49 (49)	NA
T-A-T-A-T-A	51 (51)	NA
**25% tumour reduction at cycle 1**		
< 25%	43 (43)	15 (48)
≥ 25%	57 (57)	16 (52)
**Pathological lymph node involvement ^a^**		
No	31 (41)	7 (24)
Yes	44 (59)	22 (76)

a 75 and 29 patients in the primary and validation cohort underwent surgery respectively; NA: not applicable.

### Expression of stromal proteins in tumor versus adjacent normal tissue

**Figure 1:**
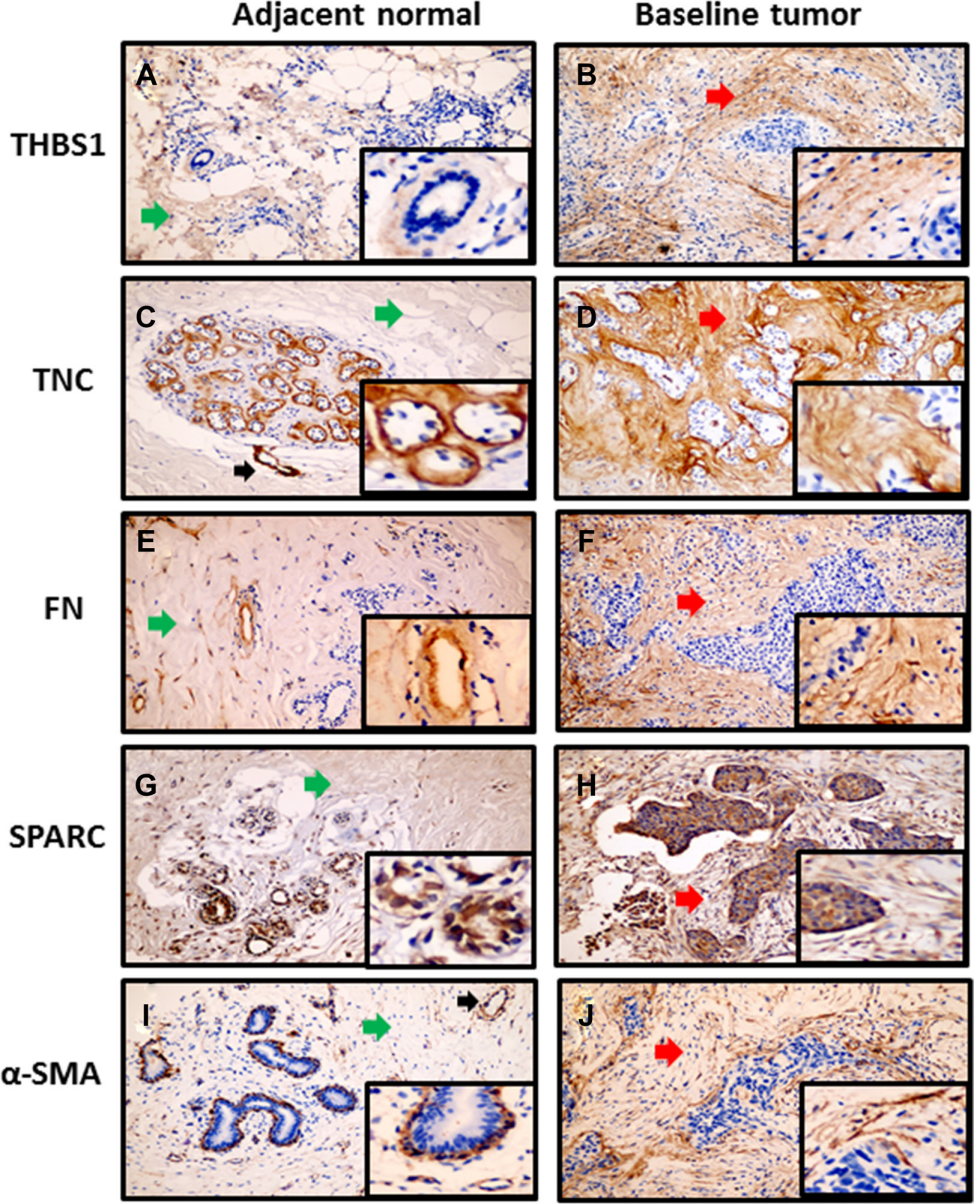
Immunoreactivity of stromal proteins in baseline tumor with matched adjacent normal tissue Magnification 100× and 400× (inserted pictures). (**A, C, E, G, and I**) Weak stromal proteins expression in the stroma area (green arrows) of adjacent normal tissue. Black arrows in panel C and panel I showed positive staining in blood vessels. (**B**, **D**, **F**, **H** and **J**) Moderate to strong stromal proteins expression in the stroma area (red arrows) of matched baseline tumor.

Full sections from baseline breast tumor specimens from the primary study cohort containing adjacent normal tissue were cut. Immunostaining was performed with the relevant primary antibodies. In the adjacent normal tissue, immunostaining of stromal proteins was detected on the blood vessels (TNC, FN and α-SMA), basement membrane (TNC), myoepithelial (α-SMA) and luminal (SPARC) cells of mammary glands respectively (Figure 1A, 1C, 1E, 1G, 1I). In contrast, expression of these proteins was predominately found in the area of intratumoral stroma, apart from concordant immunostaining in cancer cells for SPARC (Figure 1B, 1D, 1F, 1H, 1J).

### High expression of stromal proteins in both baseline and post-treatment tumors was associated with poor survival in the primary cohort (Figure 2 and Table 2)

**Figure 2:**
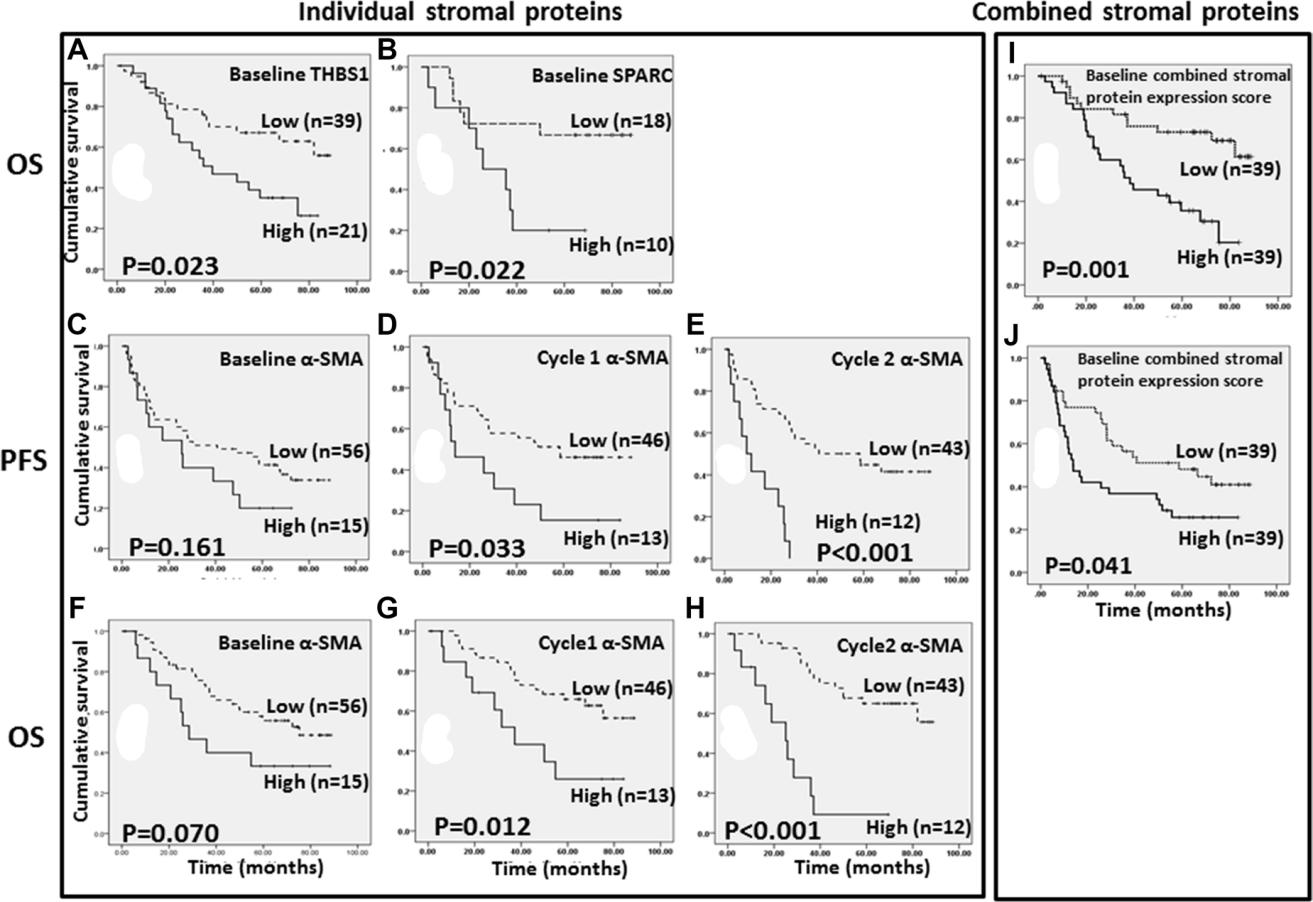
Association between (**A**) baseline THBS1 expression and overall survival; (**B**) baseline SPARC expression and overall survival; (**C–E**) α-SMA expression at baseline, cycle 1 and cycle 2 with progression-free survival; (**F–H**) α-SMA expression at baseline, cycle 1 and cycle 2 with overall survival; (**I**) baseline combined stromal protein expression score and overall survival; (**J**) baseline combined stromal protein expression score and progression-free survival.

**Table 2:** Univariate and multivariate analysis of correlation between baseline stromal proteins expression and progression-free survival and overall survival

Stromal proteins	*N*^a^	PFS				OS			
		Crude HR (95% CI)	p^c^	Adjusted HR (95% CI)^b^	p^c^	Crude HR (95% CI)	p^c^	Adjusted HR (95% CI)^b^	p^c^
**THBS1**									
Low	39	1.00 (Ref.)		1.00 (Ref.)		1.00 (Ref.)		1.00 (Ref.)	
High	27	1.45 (0.79–2.65)	NS	0.95 (0.46–2.02)	NS	2.27 (1.12–4.60)	***0.023***	2.06 (0.71–5.99)	NS
Unknown	29	1.17(0.63–2.16)	NS	1.12 (0.47–2.66)	NS	1.50 (0.71–3.16)	NS	2.92 (1.12–7.67)	NS
**TNC**									
Low	43	1.00 (Ref.)		1.00 (Ref.)		1.00 (Ref.)		1.00 (Ref.)	
High	36	1.47 (0.85–2.56)	NS	1.12 (0.55–2.27)	NS	1.72 (0.92–3.20)	NS	1.38 (0.60–3.17)	NS
Unknown	16	1.03 (0.60–2.14)	NS	1.41 (0.52–3.85)	NS	0.93 (0.37–2.34)	NS	0.63 (0.18–2.21)	NS
**FN**									
Low	30	1.00 (Ref.)		1.00 (Ref.)		1.00 (Ref.)		1.00 (Ref.)	
High	50	0.99 (0.56–1.74)	NS	1.10 (0.48–2.49)	NS	1.02 (0.53–1.97)	NS	1.87 (0.65–5.39)	NS
Unknown	15	1.03 (0.48–2.20)	NS	1.05 (0.35–3.20)	NS	1.25 (0.53–2.99)	NS	0.98 (0.25–3.81)	NS
**SPARC**									
Low	18	1.00 (Ref.)		1.00 (Ref.)		1.00 (Ref.)		1.00 (Ref.)	
High	11	1.65 (0.66–4.12)	NS	1.56 (0.55–4.43)	NS	3.52 (1.20–10.33)	***0.022***	3.78 (1.03–13.92)	***0.045***
Unknown	66	1.13 (0.58–2.20)	NS	1.05 (0.44–2.50)	NS	1.75 (0.73–4.21)	NS	1.22 (0.39–3.87)	NS
**α-SMA**									
Low	56	1.00 (Ref.)		1.00 (Ref.)		1.00 (Ref.)		1.00 (Ref.)	
High	15	1.49 (0.78–2.89)	NS	0.67 (0.28–1.58)	NS	1.83 (0.88–3.81)	NS	2.17 (0.86–5.50)	NS
Unknown	24	0.84 (0.45–1.59)	NS	1.05 (0.48–2.27)	NS	0.93 (0.45–1.94)	NS	1.97 (0.80–4.83)	NS
**Baseline combined stromal protein expression score^f^**									
Low	39	1.00 (Ref.)		1.00 (Ref.)		1.00 (Ref.)		1.00 (Ref.)	
High	39	1.79 (1.02–3.14)	***0.044***	2.22 (1.06–4.64)	***0.034***	3.17 (1.56–6.44)	***0.001***	5.94 (2.25–15.71)	***< 0.001***

a 5 baseline core biopsies without tumor presence were excluded;

b adjusted for age, tumor grade, metastasis, tumor size, lymph node involvement, ER status, PR status and Her2 status;

c Cox regression models analysis;

Ref: reference; NS: not significant.

Immunoreactivity of stromal proteins was scored by two breast pathologists (W.T and S.S) independently and grouped into low versus high expression subgroups using the cut-offs shown in Supplementary Table S1. Kappa values for inter-observer agreement on IHC scoring were 0.950 (THBS1), 0.920 (TNC), 0.958 (FN), 0.924 (SPARC), and 0.909 (α-SMA) respectively. High baseline THBS1 and SPARC were significantly associated with poorer OS (Figure 2A–2B), and high baseline SPARC was an independent prognostic marker in multivariate analysis (HR_adjusted_ 3.78, 95% CI 1.03–13.92, *p* = 0.045), adjusted for age, tumor grade, metastasis, tumor size, pathological lymph node involvement, ER, PR and Her2 status. While high baseline α-SMA expression only showed a trend in association with both shorter PFS and OS (Figure 2C, 2F), high α-SMA in post-treatment tumors following 1 cycle of chemotherapy was significantly associated with both shorter PFS (mean PFS 29.0 [95% CI 14.3–43.7] vs 52.4 [95% CI 42.9-63.0] months for high vs low α-SMA, *p* = 0.033, Figure 2D) and OS (mean OS 43.0 [95% CI 27.2–58.7] vs 67.5 [95% CI 52.9–75.8] months for high vs low α-SMA, *p* = 0.012, Figure 2G). Similarly, high α-SMA in post-treatment tumors following 2 cycles of chemotherapy remained significantly associated with both shorter PFS (mean PFS 13.7 [95% CI 8.10–19.2] vs 51.2 [95% CI 40.6–61.8] months for high vs low α-SMA, *p* < 0.001, Figure 2E) and OS (mean OS 25.6.0 [95% CI 15.4–35.9] vs 69.5 [95% CI 61.4–77.6] months for high vs low α-SMA, *p* < 0.001, Figure 2H). Expression of the other stromal proteins after chemotherapy was not associated with either PFS or OS.

The IHC staining of 5 stromal proteins for the primary cohort was performed on the TMA tissue blocks constructed from core biopsies from each patient. In some cases, there was not sufficient tissue for IHC staining of all 5 stromal proteins. Thus, we were unable to obtain IHC data for all 5 stromal proteins in these cases. To study the combined effect of intratumoral stromal proteins on PFS and OS, patients with baseline expression scores of at least 3 stromal proteins (*n* = 78) in the primary cohort were selected to construct a combined score model for intratumoral stromal proteins. High baseline combined expression score for stromal proteins was significantly associated with both shorter PFS (mean PFS 33.9 [95% CI 23.6–44.2] vs 52.3 [95% CI 41.4–63.2] months for high vs low combined stromal protein expression score, *p* = 0.041, Figure 2J) and OS (mean OS 45.6 [95% CI 36.4–54.8] vs 70.2 [95% CI 60.0–79.5] months for high *vs* low combined stromal protein expression score, *p* = 0.001, Figure 2I). In multivariate analysis, high baseline combined stromal protein expression score was an independent predictor for both poorer PFS (HR_adjusted_ 2.22, 95% CI 1.06–4.64, *p* = 0.034) and OS (HR_adjusted_ 5.94, 95% CI 2.25–15.71, *p* < 0.001).

### Stromal proteins expression increased after chemotherapy in the primary cohort

**Figure 3:**
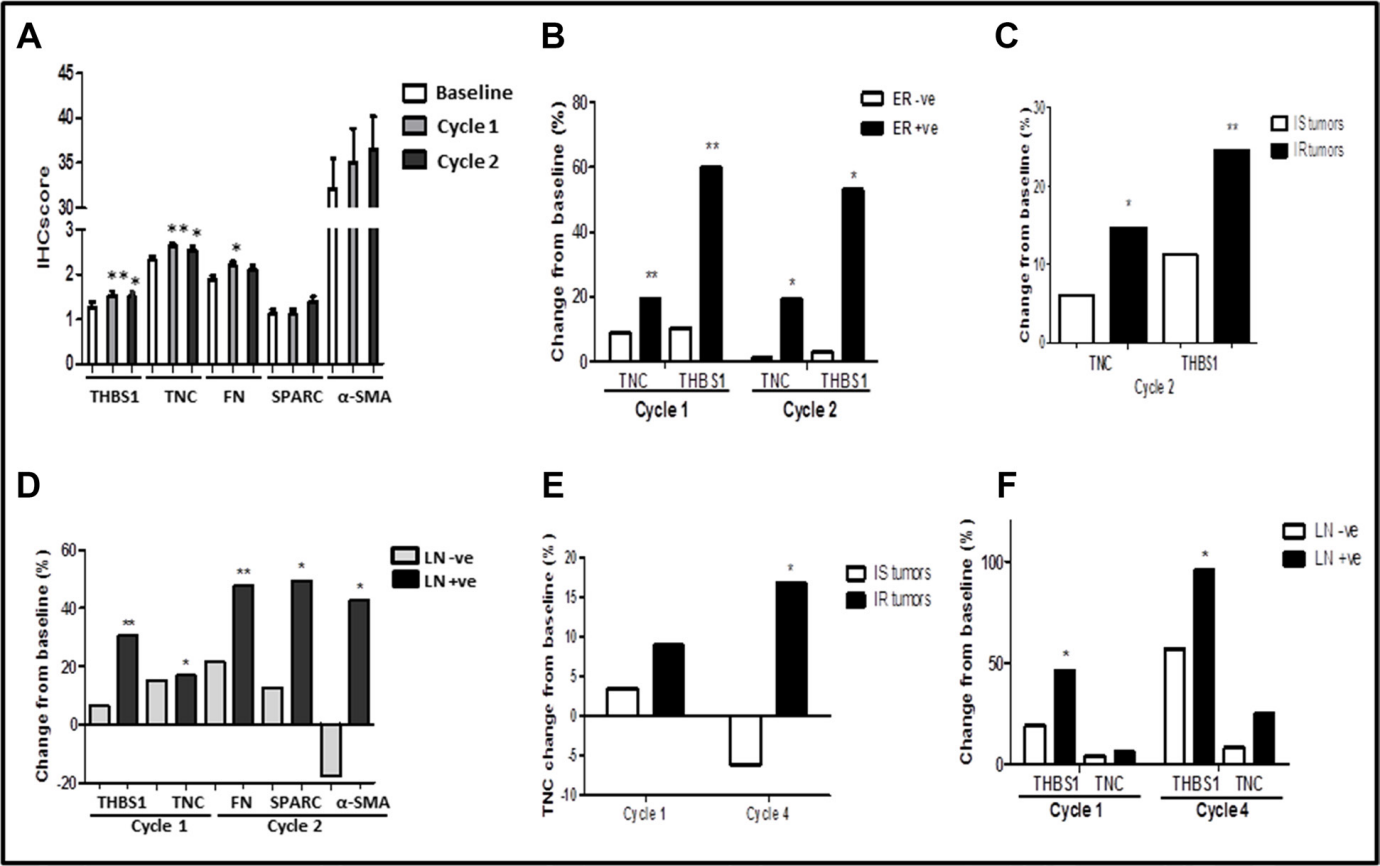
(**A**) Mean expression of THBS1, TNC, FN, SPARC and α-SMA at baseline, after cycle 1 and after cycle 2 chemotherapy in the entire primary study cohort; (**B**) Comparison of changes in THBS1 and TNC expression after cycle 1 and 2 chemotherapy relative to baseline in estrogen receptor (ER) positive and negative subgroups in the primary cohort; (**C**) Comparison of changes in THBS1 and TNC after cycle 2 chemotherapy relative to baseline in intrinsically sensitive (IS) and resistant (IR) tumors in the primary cohort; (**D**) Comparison of changes in THBS1, TNC, FN, SPARC and α-SMA expression after 1–2 cycles of chemotherapy relative to baseline in pathological lymph node (LN) positive and negative subgroups in the primary cohort; (**E**) Changes in TNC expression after cycle 1 and cycle 4 chemotherapy relative to baseline in the validation cohort in patients with intrinsically sensitive (IS) and resistant (IR) tumors; (**F**) Changes in THBS1 and TNC expression after cycle 1 and cycle 4 chemotherapy relative to baseline in the validation cohort in patients with or without LN involvement. * *p* < 0.05, ** *p* < 0.01

Concordant with previous findings [14], baseline expression in TNC and FN showed mild positive association (Spearman correlation = 0.324, *p* = 0.005). No further associations were found between the expression of the 5 stromal proteins both at baseline and after chemotherapy. Figure 3A showed the IHC expression alterations of THBS1 (*n* = 66), TNC (*n* = 79), FN (*n* = 80), SPARC (*n* = 62) and α-SMA (*n* = 50) after the first and second cycle of chemotherapy. There was statistically significant up-regulation of THBS1 and TNC expression after both cycle 1 and cycle 2 chemotherapy compared with baseline (mean THBS1 1.27 ± 0.92, 1.52 ± 0.88, 1.50 ± 0.85 for baseline, cycle 1 and cycle 2, *p* = 0.008 for cycle 1 vs baseline, *p* = 0.019 for cycle 2 vs baseline; mean TNC 2.32 ± 0.71, 2.63 ± 0.57, 2.54 ± 0.72 for baseline, cycle 1 and cycle 2, *p* = 0.001 for cycle 1 vs baseline, *p* = 0.037 for cycle 2 vs baseline). FN expression increased significantly after 1 cycle of chemotherapy (mean 1.88 ± 0.79 vs 2.19 ± 0.76 for baseline vs cycle 1, *p* = 0.041). Taken together, chemotherapy was observed to significantly increase stromal proteins expression (THBS1, TNC and FN) and in particular, induce persistent increased expression of THBS1 and TNC compared with FN, suggesting that THBS1 and TNC may play more important roles in regulating response to doxorubicin- and docetexal-based chemotherapy.

### Chemotherapy-induced expression changes of stromal proteins in relation to estrogen receptor status

At baseline, there was no significant difference in the expression levels of all 5 stromal proteins between ER-negative and -positive tumors (Supplementary Table S2). However, significant increase in the expression of THBS1 and TNC was observed after the first and second chemotherapy cycle in ER-positive tumors (THBS1 change 60.00% and 53.13%, *p* = 0.001 and *p* = 0.012; TNC change 19.42% and 19.29%, *p* = 0.006 and *p* = 0.021 for cycle 1 or cycle 2 vs baseline respectively; Figure 3B). In contrast, no significant changes were seen in THBS1 and TNC expression in ER-negative tumors (Supplementary Table S3). No significant differences in chemotherapy-induced expression changes were observed for FN, SPARC and α-SMA between ER-positive and -negative tumors.

### Increased expression of stromal proteins with chemotherapy in intrinsically resistant tumors

Baseline expression of all 5 stromal proteins was not significantly different between intrinsically sensitive (IS) and resistant (IR) tumors (Supplementary Table S2). However, following two cycles of chemotherapy, increased expression of both THBS1 and TNC was observed in IR tumors (mean THBS1 1.30 ± 0.75 vs 1.62 ± 0.82, change 24.62%, *p* = 0.007; mean TNC 2.31 ± 0.63 vs 2.65 ± 0.61, change 14.72%, *p* = 0.017; Figure 3C). In contrast, no significant up-regulation in THBS1 and TNC was seen in patients with IS tumors (mean THBS1 1.25 ± 1.05 vs 1.39 ± 0.87, *p* = 0.432; mean TNC 2.32 ± 0.77 vs 2.46 ± 0.79, *p* = 0.385). No significant differences in chemotherapy-induced expression changes in FN, SPARC and α-SMA were observed between IS and IR tumors.

### Up-regulation of stromal proteins following 1–2 cycles of neoadjuvant chemotherapy correlated with subsequent pathological lymph node involvement at surgery

Baseline expression of stromal proteins did not differ significantly between patients with or without PLN involvement at surgery, except for FN, whose basal expression was higher in patients without PLN involvement (*p* = 0.036; Supplementary Table S2). However, after 1–2 cycles of chemotherapy, patients who had PLN involvement at surgery showed significant up-regulation of tumor THBS1, TNC, FN, SPARC and α-SMA expression (Figure 3D). After one cycle of chemotherapy, there was significant THBS1 and TNC up-regulation in relation to baseline (mean THBS1 1.25 ± 0.93 vs 1.63 ± 0.88, change 30.40%, *p* = 0.003; mean TNC 2.18 ± 0.79 vs 2.55 ± 0.55, change 16.97%, *p* = 0.015) compared to patients without PLN involvement (mean THBS1 1.25 ± 1.00 vs 1.33 ± 0.86, change 6.40%, *p* = 0.166; mean TNC expression 2.30 ± 0.57 vs 2.65 ± 0.57, change 15.22%, *p* = 0.083). Similarly, compared to baseline, up-regulation of FN, SPARC and α-SMA following 2 cycles of chemotherapy was observed in tumors from patients with PLN involvement (mean FN 1.66 ± 0.80 vs 2.45 ± 0.77, change 47.59%, *p* = 0.005; mean SPARC 0.94 ± 0.77 vs 1.40 ± 1.10, change 49.33%, *p* = 0.046; mean α-SMA 25.8 ± 27.4 vs 36.8 ± 29.7, change 42.64%, *p* = 0.021). In contrast, there were no significant chemotherapy-induced tumor expression changes in FN, SPARC and α-SMA in patients without PLN involvement (mean FN 2.10 ± 0.72 vs 2.55 ± 0.76, change 20.85%, *p* = 0.284; mean SPARC 1.23 ± 0.86 vs 1.39 ± 0.98, change 12.70%; mean α-SMA 36.90 ± 31.80 vs 30.30 ± 25.20, change −17.89%, *p* = 0.205). These results suggest that increase in expression of THBS1, TNC, FN, SPARC and α-SMA early in the course of neoadjuvant chemotherapy may be predictive of PLN metastasis and hence poorer clinical outcomes; both THBS1 and TNC may be better predictors than FN, SPARC and α-SMA as changes occurred earlier.

### Validation of chemotherapy-induced expression changes in THBS1 and TNC in an independent cohort

In the primary cohort, we have shown, among 5 stromal proteins, that both THBS1 and TNC were more related with chemotherapy resistance, including intrinsic resistance and lymph node involvement at surgery. Therefore, we further validated the prognostic and predictive values of THBS1 and TNC in an independent cohort. Although we did not find significant association between the baseline and post-treatment levels of these two stromal proteins with either PFS or OS, both THBS1 and TNC expression showed trends for progressive increase from baseline, cycle 1 to cycle 4 (mean THBS1 1.05 ± 0.94, 1.30 ± 0.89, 1.70 ± 0.66, *p* = 0.020; mean TNC 2.11 ± 0.81, 2.21 ± 0.63, 2.42 ± 0.77, *p* = 0.232), consistent with what was observed in the primary cohort. Similarly, patients with IR tumors had significant increase in TNC expression in post-cycle-4 tumor specimens compared to baseline (mean 2.14 ± 0.66, 2.33 ± 0.62, 2.50 ± 0.80 for baseline, cycle 1 and cycle 4; change 8.89% and *p* = 0.102 for cycle 1 vs baseline; change 16.67% and *p* = 0.034 for cycle 4 vs baseline; Figure 3E). Although the significant increase in THBS1 was not obvious in IR tumors, patients with PLN had significant increase in tumor THBS1 expression in both the post-cycle-1 and post-cycle-4 tumor specimens compared to baseline (mean THBS1 0.72 ± 0.83, 1.06 ± 0.80, 1.41 ± 0.62 for baseline, cycle 1 and cycle 4; change 46.16% and *p* = 0.034 for cycle 1 vs baseline; change 95.49% and *p* = 0.032 for cycle 4 vs baseline; Figure 3F). An increasing trend was observed in TNC expression in patients with pathologically involved lymph nodes (change 8.33% for cycle 1 vs baseline; change 25% for cycle 4 vs baseline) although the difference was not statistically significant (Figure 3F). Taken together, both THBS1 and TNC expression changes in relation to chemotherapy response in this independent dataset were consistent with our findings in the primary cohort and confirmed their association with chemo-resistance.

### THBS1 and TNC protected MCF-7 cells against proliferation inhibition induced by docetaxel through activating integrin β1/mTOR pathway

**Figure 4:**
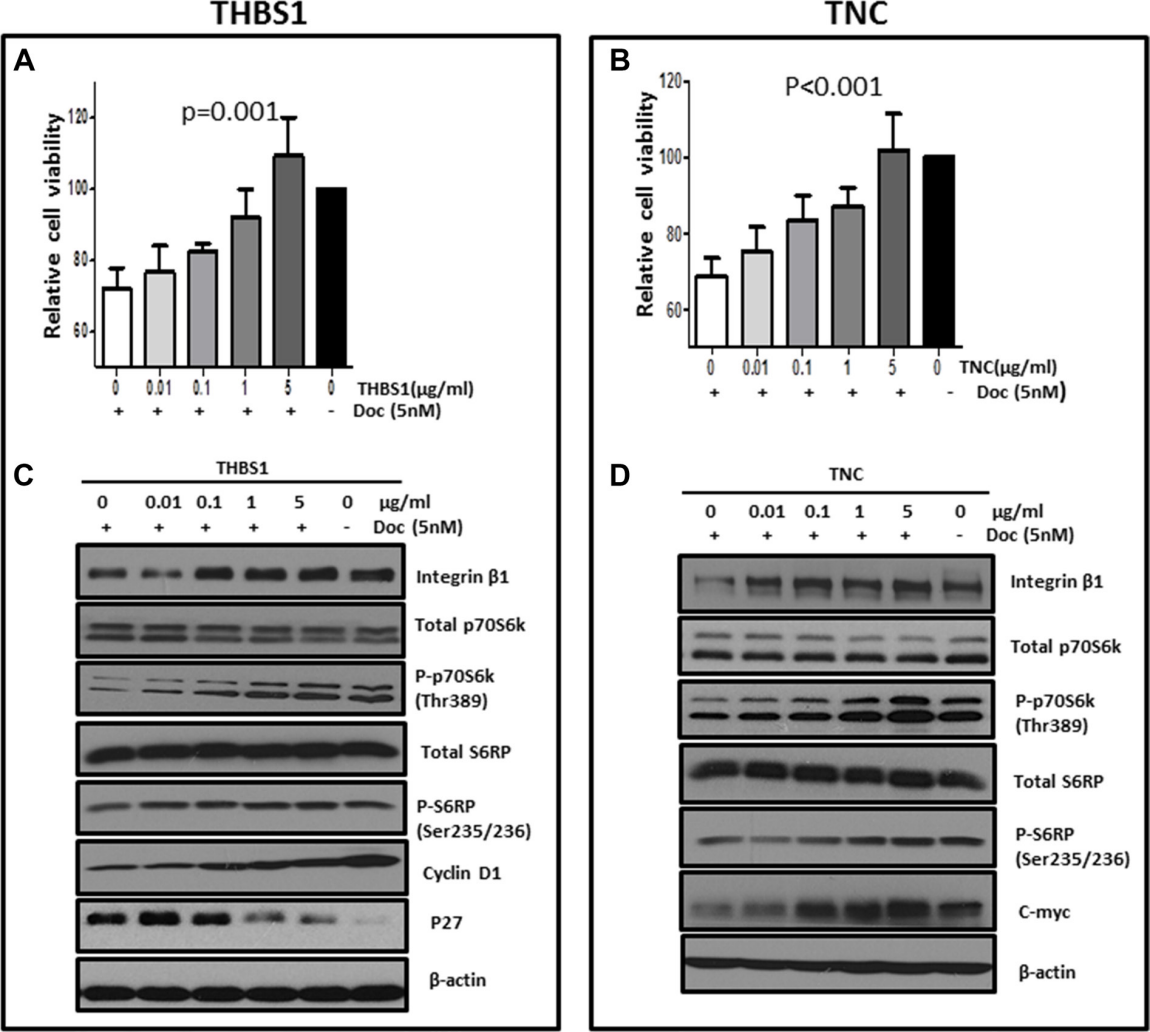
Exogenous THBS1 and TNC protected MCF-7 cells against proliferation inhibition by docetaxel through activating integrin β1/mTOR pathway and deregulating cell cycle proteins MCF-7 cells grew in DMEM containing 5% CS-FBS for 48 hours. Then recombinant protein THBS1 or TNC at indicated concentration (0, 0.01, 0.1, 1 and 5 μg/ml) was added into the media respectively and maintained for 48 hours, with the presence or absence of 5 nM of docetaxel. MTS assay was performed and the protective effects of THBS1 (**A**) or TNC (**B**) were analyzed using one-way ANOVA, compared with groups treated with docetaxel alone. The effects of THBS1 (**C**) and TNC (**D**) on biomarkers for mTOR pathway and cell cycle were further evaluated using Western blots analysis.

By studying serial breast cancer specimens collected during chemotherapy from two prospective clinical trials, we have shown that both THBS1 and TNC were associated with docetaxel treatment resistance, either administered sequentially with doxorubicin in the primary cohort, or as a single agent in the validation cohort. Docetaxel is an active chemotherapeutic agent that is commonly used in both early-stage and advanced breast cancer. Therefore, we went on to determine the underlying mechanisms by which THBS1 or TNC protects breast cancer cells from the cytotoxic effects of docetaxel. After 48 hours treatment with recombinant THBS1 and TNC in the presence or absence of docetaxel respectively, the protective effects of THBS1 and TNC on MCF-7 cells were measured by MTS assay and Western blot analysis. As shown in Figure 4, 5 nM of docetaxel treatment caused around 30% arrest of cell growth, compared with vehicle controls. Exogenous THBS1 and TNC were able to rescue the growth of MCF-7 cells in a dose dependent manner (*p* = 0.001 for THBS1, Figure 4A; *p* < 0.001 for TNC, Figure 4B). At concentrations of 5 μg/ml, compared with docetaxel-treated groups, THBS1 and TNC rescued MCF-7 cell growth by 50% and 40% respectively, which were close to the viability rate of individual vehicle control. Furthermore, immunoblotting analysis showed that docetaxel treatment led to cell cycle arrest by decreasing cyclin D1 and c-myc and by increasing tumor suppression gene p27. THBS1 treatment reversed docetaxel-induced cell cycle arrest by enhancing cyclin D1 and suppressing p27 expression (Figure 4C). In contrast, TNC blocked the inhibitory effects of docetaxel on cell cycle by increasing c-myc expression (Figure 4D). Therefore, it appears that THBS1 and TNC promote breast cancer cell proliferation through differential mechanisms. As for the involvement of integrin β1/mTOR, docetaxel treatment decreased the levels of integrin β1 and deactivated mTOR signaling by dephosphoylating both p70S6 at Thr389 and S6RP at Ser235/236. Both THBS1 and TNC restored integrin β1 expression and activated mTOR pathway by phosphorylating p70S6K and S6RP in a dose dependent manner (Figure 4C–4D). Taken together, we confirmed that THBS1 and TNC protected MCF-7 cells from docetaxel cytotoxicity through activating integrin β1/mTOR pathway and deregulating cell cycle proteins.

## DISCUSSION

There is an urgent clinical need to identify biomarkers that can be used to predict which patients will benefit most from cytotoxic chemotherapy. More recently, it has been recognised that the intra-tumoral stroma contributes significantly to most of the ‘hallmarks’ of cancer. Tumors with a high stroma content had an increased hazard for tumor relapse independent of other clinicopathological parameters in node-negative premenopausal breast cancer patients who received one course of perioperative doxorubicin-based chemotherapy [15]. Furthermore, alterations in expression patterns of stromal genes have been shown to predict resistance to pre-operative anthracyclines-containing chemotherapy in breast cancer [3]. In this study, we showed that at the protein level, high baseline THBS1 and SPARC and high post-treatment α-SMA expression were associated with poor survival; a high baseline score derived from the combined expression of 5 stromal proteins was an independent predictor for poor progression-free and overall survival. Increased expression of THBS1 and TNC following chemotherapy occurred in intrinsically resistant tumors, while early up-regulation of THBS1, TNC, FN, SPARC and α-SMA following neoadjuvant chemotherapy was associated with subsequent pathological lymph node involvement at surgery. The association of chemotherapy-induced up-regulation of THBS1 and TNC with chemo-resistance was reproduced in an independent patient cohort. Through functional studies, we confirmed that both THBS1 and TNC protected MCF-7 cells from proliferation inhibition induced by docetaxel through activating integrin β1/mTOR pathway and deregulating cell cycle proteins.

Cancer-associated fibroblasts and extracellular matrix are the main components of the intratumoral stroma, which is critical in promoting cell motility and thus promoting breast cancer progression [16, 17]. α-SMA, TNC and SPARC, which represents the abundance of cancer-associated fibroblasts and extracellular matrix, have been linked to shorter survival in breast cancer in retrospective studies based on their gene or protein expression levels [10, 11, 13, 18]. In this prospective neoadjuvant clinical trial, we confirmed that high expression of baseline SPARC and post-treatment α-SMA was associated with shorter survival. Moreover, previous studies showed that cancer-associated stroma in invasive breast cancer independently predicted tumor recurrence, distant metastasis and subsequent poor clinical outcomes [4, 19] and, in particular, a panel of 26 stroma-derived genes could forecast disease outcome [4]. In our study, we showed that a combined high expression score of 5 stromal proteins at baseline predicted for poor PFS and OS independently. These results strongly suggest that these 5 stromal proteins play a crucial role in prognosticating breast cancer and that a combined stromal score model may be more useful to predict prognosis of breast cancer patients.

It was reported that up-regulation of stroma-related genes followed epirubicin- or taxane-based neoadjuvant chemotherapy [20, 21], suggesting that stroma-related genes may play important roles in response or in mediating resistance to chemotherapy. In the context of doxorubicin- and docetaxel-based neoadjuvant chemotherapy, we showed predominant up-regulation in THBS1, TNC and FN after 3 weeks of chemotherapy when compared with matched baseline specimens; the increase persisted after 6 weeks of chemotherapy for THBS1 and TNC, particularly in ER-positive tumors. The more marked increased expression of stromal proteins following chemotherapy in ER-positive tumors may be associated with ER-stimulated differentiation of fibroblasts into myofibroblasts and formation of extracellular matrix [22, 23]. As shown in a previous study, THBS1 was directly stimulated by estrogens in ER-positive breast cancer cells [24]. Taken together, our results suggest that THBS1 and TNC may be the more important stromal proteins that respond to chemotherapy and thus may play crucial roles in regulating chemoresponse.

Several studies have suggested that stromal gene expression levels may be important clinical indicators of tumor response to either combined or single agent neoadjuvant chemotherapy in breast cancer. For example, Farmer et al. [3] showed that increased stromal gene expression in reactive stroma in breast cancer predicted resistance to preoperative chemotherapy with 5-fluorouracil, epirubicin and cyclophosphamide. Azim et al [25] further confirmed an association between high SPARC mRNA expression and low pathological complete response rate especially in the Her2-subtype breast tumors following neoadjuvant anthracycline with or without taxanes. An earlier result showed that THBS1 promoted breast cancer to metastasize to lungs in the polyomavirus middle T antigen transgenic mouse, suggesting that THBS1 plays a role in mammary cancer cell migration [26]. We found that tumors with pathological lymph node involvement at surgery following neoadjuvant chemotherapy had significant increase in THBS1 expression after one cycle of chemotherapy, which was independently confirmed in a second cohort. In concordance, increased expression of THBS1 following chemotherapy was also observed in intrinsically resistant tumors. Although the role of THBS1 in regulating tumor progression was controversial [27–29], in invasive cancer, THBS1 may function as an adhesive protein or a modulator of extracellular proteases to promote tumor invasion [26, 30].

TNC on the other hand, plays a major role in promoting cell migration by remodeling cancer-associated stroma [31] and activating integrin pathway [32] and has thus been associated with local and distant recurrence [33]. Of note, breast cancer cell-derived TNC was essential in initiation and promotion of the outgrowth of pulmonary micrometastases [34], and high TNC expression was associated with treatment resistance to tamoxifen [10]. TNC protein expression was up-regulated in breast cancer patients with progressive disease treated with either anthracyclin- or taxane-based monotherapy [21]. In our study, statistically significant up-regulation of TNC expression after 1-2 cycles of chemotherapy was observed in patients with intrinsically resistant tumors as well as those with pathological lymph node involvement. Enhanced regulation of cancer stem cell population may partially explain TNC-associated resistance to conventional chemotherapy [34, 35]. In addition, concordant with previous findings [36, 37], increase in expression of FN, SPARC and α-SMA after two cycles of chemotherapy was associated with subsequent pathological lymph node involvement, and these stromal proteins may also promote metastasis through a stroma-remodeling manner [14, 17]. Collectively, increases in expression of stromal proteins a few weeks after chemotherapy were associated with treatment resistance and have the potential to serve as biomarkers to stratify breast cancer patients into distinct chemoresponse subgroups. These suggest that stromal proteins could be used as predictive biomarkers of resistance to common chemotherapy drugs currently administered in breast cancer patients.

Both THBS1 and TNC are well-known extracellular matrix (ECM) proteins, which activate integrin signaling pathway in mammal cells. It is less clear, however, what roles THBS1 and TNC-associated signaling have in regulation of chemotherapy resistance in breast cancer. We found that both THBS1 and TNC rescued MCF-7 cells from docetaxel-induced proliferation arrest through activating integrin β1/mTOR pathway and deregulating cell cycle progression. THBS1 and TNC in the tumor microenvironment may bind to and activate integrin β1 on the surface of breast cancer cells to phosphorylate intracellular mTOR pathway. Activated mTOR signaling in turn promotes the transcription and translation of its downstream effectors cyclin D1 and c-myc as well as degrades p27 to promote cell cycle G1/S progression. Our findings are consistent with a previous report which identified integrin β1/Akt signaling as an important survival pathway in paclitaxel-induced apoptosis in breast cancer cells [42]. Taken together, our results identify THBS1- or TNC-activated integrin β1/mTOR signaling respectively as an important survival pathway in chemotherapy-induced growth inhibition in breast cancer cells and suggest that activation of this pathway may contribute to the development of chemotherapy resistance. Therefore, targeting ECM/integrin β1/mTOR pathway may be a promising therapeutic strategy to overcome chemotherapy resistance in breast cancer. Currently, several integrin inhibitors such as a humanized anti-β1 antibody are being tested in clinical trials as therapeutic agents for cancer [43]. In addition, mTOR inhibitors have been found to be additive or synergistic with both chemotherapy and endocrine therapy [44, 45]. Randomized phase 3 clinical trials have confirmed the therapeutic effects of everolimus, an mTOR inhibitor, in breast cancer patients. For instance, everolimus combined with an aromatase inhibitor improved progression-free survival in patients with hormone-receptor-positive advanced breast cancer compared to an aromatase inhibitor alone [46]. Addition of everolimus to trastuzumab plus vinorelbine significantly prolonged progression-free survival in patients with trastuzumab-resistant and taxane-pre-treated, Her2-positive, advanced breast cancer [47]. As yet, no reliable predictive biomarkers have been identified to select patients most likely to benefit from an mTOR inhibitor. Our findings suggest that CAF proteins may be potential biomarkers in breast cancer for response to mTOR inhibition, and further studies may be performed to evaluate this.

## CONCLUSIONS

A high combined expression score of 5 stromal proteins, namely THBS1, TNC, FN, SPARC and α-SMA, in baseline untreated breast cancers, is associated with shorter survival, while their up-regulation after chemotherapy predicted for poor treatment response. This suggests that this panel of stromal proteins not only could be used as prognostic biomarkers to stratify breast cancer patients into distinct subgroups of clinical outcomes but also could be potential predictive biomarkers for chemotherapy response. Moreover, we found that both THBS1- and TNC-activated integrin β1/mTOR signaling played a role in regulating chemotherapy resistance, suggesting that targeting integrin β1/mTOR pathway may be a promising therapeutic strategy to overcome chemotherapy resistance in breast cancer.

## MATERIALS AND METHODS

### Primary study cohort and definition of treatment outcomes

100 female patients with newly diagnosed locally advanced or metastatic breast cancer were recruited into a prospective phase II study and randomized to one of two alternating sequences of doxorubicin 75 mg/m^2^ (A) and docetaxel 75 mg/m^2^ (T) every three weeks for six cycles, followed by breast cancer surgery. The institutional ethics committee approved the study protocol, and all patients provided written informed consent. Patients were classified as having intrinsically sensitive (IS) or resistant (IR) tumors to the chemotherapy they received in the first cycle if they achieved ≥ 25% or < 25% reduction in tumor dimensions, respectively, after the first chemotherapy cycle. The presence of cancer cells in axillary lymph nodes under microscopic examination after definitive surgery was defined as pathological lymph node involvement at surgery. Progression-free survival (PFS) and overall survival (OS) were defined as the time between the date of randomization and the first documented evidence of progression (PFS) or death (OS) respectively, or the last follow-up whichever came first.

### Validation cohort

31 breast cancer patients treated with four cycles of neoadjuvant docetaxel in another clinical trial was used as a validation set (ClinicalTrials.gov identifier: NCT00212095). In brief, pre-, post-cycle-1- and post-cycle-4- chemotherapy tumor core biopsies were obtained. Intrinsic sensitivity to docetaxel was defined using the same response criteria as in the primary study cohort.

### Immunohistochemistry (IHC) analysis and IHC scoring

As described previously [38, 39], 3 serial tumor core biopsies were taken from patients in the primary study cohort; pre-, post-cycle-1-, and post-cycle-2-treatment respectively. Tissue microarray (TMA) was constructed using a tissue arrayer ATA-100 (Chemicon, USA) and 4 μm-thickness sections were cut. Full sections from 30 cases of baseline breast tumor specimens from the primary study cohort containing adjacent normal tissue, and from the serial biopsies of the validation cohort were also cut. Immunostaining was performed with the relevant primary antibodies as described previously [39]. Immunoreactivity was independently scored by two pathologists (T.W and S.S). The immunostaining intensity of THBS1, TNC, FN and SPARC [36, 40] was scored as 0 to 3 for negative, mild, moderate and strong staining respectively. Scoring for α-SMA was determined by assessing percentage of positive staining [41] as described previously. Antibodies, manufacturers, dilutions, IHC scoring criteria and cut-offs for determining low versus high expression were shown in Supplementary Table S1. Change in stromal proteins expression from baseline after chemotherapy was calculated as follows: change from baseline (%) = 100% × (mean expression post-treatment – mean expression at baseline)/mean expression at baseline.

### Survival model construction using intratumoral stromal protein expression

The IHC staining of 5 stromal proteins for the primary cohort was performed on the TMA tissue blocks constructed from core biopsies from each patient. In some cases, core tissue in TMA blocks was not sufficient for IHC staining of all 5 stromal proteins. Therefore, IHC data for certain stromal proteins in these cases were missing. To study the combined effect of intratumoral stromal proteins on PFS and OS, patients with baseline IHC data from at least 3 of the 5 stromal proteins (*n* = 78) in the primary cohort were selected to construct a score model for intratumoral stroma proteins. The expression levels for individual stromal protein were assigned as three levels; high expression, low expression and missing. Each individual stromal protein was modeled separately using a Cox regression model adjusted for age, tumor grade, metastasis, tumor size, pathological lymph node involvement, ER, PR and Her2 status, in order to generate an adjusted effect of the stromal protein on the outcome of interest. From these models the regression coefficients for each protein – high, low and missing was extracted and subsequently used to create a combined stromal protein expression score by summing up all coefficients for each patient. Each patient was then categorized into a combined high and low stromal protein expression group based on the median value of the summed coefficients. This score was entered into a final multivariate Cox regression testing the independent effect of high versus low combined stromal protein expression score.

### Statistical analysis

Inter-observer agreement for IHC scoring was analysed using Kappa test. To determine the correlation between baseline stromal proteins expression and clinico-pathological parameters and chemotherapy response, Mann-Whitney *U* test was applied. Changes in stromal proteins expression from baseline following neoadjuvant chemotherapy were analyzed using Wilcoxon Signed Ranks Test. Serial changes of THBS1 and TNC in the validation cohort were analyzed with Friedman test. Correlations amongst stromal proteins were analysed with Spearman correlation test.

Survival analysis was conducted using Kaplan-Meier and the log-rank test was employed to compare the difference. Univariate and multivariate Cox regression models were carried out with PFS or OS as the end point. Multivariate Cox regression models were adjusted for age, tumor grade, metastasis, tumor size, pathological lymph node involvement, ER, PR and Her2/neu status. For this purpose, we entered all variables, which were univariately associated with survival, into a multivariate Cox model. Using backward stepwise selection, we eliminated variables that did not contribute significantly to the fit of the model and continued until the model consisted of variables that were significantly associated with the outcome. Protective effects of exogenous THBS1 and TNC against docetaxel in MCF-7 cells were analysed by one-way ANOVA. All statistical analyses were performed using the IBM SPSS package (version 19.0 for Windows, IBM SPSS Inc., USA) with significance set at the 5% level.

### Reagents and cell culture for cell line experiments

Docetaxel was purchased from Sigma-Aldrich Co (St. Louis, MO, USA) and dissolved in dimethyl sulfoxide (DMSO) which was used as vehicle control. Purified human THBS1 and TNC proteins were purchased from EMD Millipore Corporation (CA, USA). Charcoal stripped fetal bovine serum (CS-FBS) was obtained from Nacalai Tesque (Kyoto, Japan). Human breast cancer cell line MCF-7 (ER+) was obtained from American Type Culture Collection (ATCC, Manassas, VA, USA) and maintained in high glucose Dulbecco's Modified Eagle's Medium (DMEM) (Nacalai Tesque, Japan) supplemented with 10% fetal bovine serum (FBS) and 1X penicillin-streptomycin (Invitrogen, Carlsbad, CA) at 37°C in a humidified atmosphere with 5% CO_2_.

### Cell treatment with exogenous THBS1 or TNC

Docetaxel is clinically well-established for treatment of locally advanced or metastatic breast cancer. Therefore, to explore the protective effects of exogenous THBS1 or TNC against docetaxel, 3000 MCF-7 cells per well in 96-well plate were grown in DMEM containing 10% FBS overnight and then replaced with DMEM containing 5% CS-FBS for 48 hours. THBS1 or TNC at indicated concentrations (0, 0.01, 0.1, 1 and 5 μg/ml)) were then added into the cell culture media respectively and maintained for another 48 hours, with or without treatment with 5 nM docetaxel. DMSO was used as vehicle control for docetaxel. All experiments were performed in triplicates. Similar experiments were applied to cells seeded in 10 cm dish for Western blots analysis.

### Cell viability assay

Cell viability assays were carried out for cells treated with exogenous THBS1 or TNC by mixing with 20 μl reagent containing a tetrazolium compound [3-(4,5-dimethylthiazol-2-yl)-5-(3-carboxymethoxyphenyl)-2-(4-sulfophenyl)-2H tetrazolium, MTS] (Promega, Madison, WI). The absorbance at 490 nm was determined using a 96-well plate reader (Tecan, Männedorf, Switzerland).

### Western blots analyses

Western blots analyses were carried out with cells treated with exogenous THBS1 and TNC. Protein was separated by SDS polyacrylamide gel electrophoresis (SDS-PAGE) and transferred to polyvinylidene fluoride (PVDF) membranes (Millipore, Billerica, MA). Membranes were incubated with different primary antibodies (Supplementary Table S1). Proteins were detected by enhanced chemiluminescent immunodetection system (GE Healthcare Life Science, Little Chalfont, UK).

## SUPPLEMENTARY MATERIALS


