# Supplementary material for: High expression of intratumoral stromal proteins is associated with chemotherapy resistance in breast cancer
Source: Oncotarget. 2016 Jul 28;7(34):55155–68. doi: 10.18632/oncotarget.10894 (PMC5342408; doi:10.18632/oncotarget.10894)
Supplement: Supplementary file 1 [file oncotarget-07-55155-s001.pdf]

High expression of intratumoral stromal proteins is associated with chemotherapy resistance in breast cancer

Supplementary Materials

Supplementary Table S1: Dilutions, manufacturers for antibodies used and cut-offs for immunohistochemstry (IHC) staining

Antibodies	Clones, dilutions and Manufacturers	Cut-offs for low vs high expression
**THBS1**[a]	1:200, R&D System	Intensity 0 and 1 vs 2 and 3[b]
**TNC**[a]	1:1000, Abcam	Intensity 0 and 1 vs 2 and 3[b]
**FN**[a]	1:300, R&D System	Intensity 0 and 1 vs 2 and 3[b]
**SPARC**[a]	1:150, R&D System	Intensity 0 and 1 vs 2 and 3[b]
**α-SMA**[a]	1:400, DAKO	Positive staining ≤ 50 vs ≥ 50[c]

[a]IHC scoring on cancer-associated stroma; [b]intensity 0 to 3, reprensenting negative, mild, moderate and strong immunostaining; [c]determined by receiver-operating characteristic curve using SPSS software.

Supplementary Table S2: Relation between baseline expression of stromal proteins and estrogen receptor status, intrinsic response and pathological lymph node involvement

Stromal proteins		*N*	Mean ± SD	*P*[a]
	**Estrogen receptor status**			
**THBS1**	Negative	21	1.38 ± 0.97	0.155
	Positive	36	1.00 ± 0.83	
**TNC**	Negative	31	2.52 ± 0.68	0.065
	Positive	36	2.19 ± 0.75	
**FN**	Negative	29	1.86 ± 0.79	0.772
	Positive	37	1.81 ± 0.81	
**SPARC**	Negative	11	1.05 ± 0.86	0.159
	Positive	16	1.21 ± 0.83	
**α-SMA**	Negative	27	35.74 ± 33.50	0.52
	Positive	27	30.04 ± 27.56	
	**Intrinsic response**			
**THBS1**	Resistant	30	1.30 ± 0.75	0.73
	Sensitive	36	1.25 ± 1.05	
**TNC**	Resistant	35	2.31 ± 0.63	0.779
	Sensitive	44	2.32 ± 0.77	
**FN**	Resistant	38	1.76 ± 0.79	0.218
	Sensitive	42	1.98 ± 0.78	
**SPARC**	Resistant	14	1.04 ± 0.96	0.888
	Sensitive	14	1.17 ± 0.78	
**α-SMA**	Resistant	31	29.68 ± 27.96	0.454
	Sensitive	39	34.00 ± 31.16	
	**Pathological lymph node involvement**			
**THBS1**	Negative	16	1.25 ± 1.00	0.98
	Positive	28	1.25 ± 0.93	
**TNC**	Negative	20	2.30 ± 0.57	0.683
	Positive	34	2.18 ± 0.80	
**FN**	Negative	20	2.10 ± 0.72	0.036
	Positive	35	1.66 ± 0.80	
**SPARC**	Negative	7	0.94 ± 0.77	0.132
	Positive	9	1.23 ± 0.86	
**α-SMA**	Negative	18	36.04 ± 31.77	0.277
	Positive	32	25.78 ± 27.42	

a: Mann-Whitney *U* test.

Supplementary Table S3: Association between changes of stromal proteins following chemotherapy and tumor estrogen receptor (ER) status in the primary cohort

	BL	C1	C2	P^a (C1 vs BL)	P^a (C2 vs BL)
	Mean ± S D				
**ER positive (*n* = 37)**					
THBS1	1.00 ± 0.82	1.60 ± 0.97	1.53 ± 0.88	***0.001***	***0.012***
TNC	2.19 ± 0.74	2.62 ± 0.56	2.62 ± 0.60	***0.006***	***0.021***
FN	1.81 ± 0.81	2.11 ± 0.80	2.23 ± 0.92	0.279	0.061
SPARC	1.21 ± 0.83	1.25 ± 1.00	1.38 ± 0.98	0.979	0.182
α-SMA	31.9 ± 26.8	34.17 ± 32.69	31.91 ± 26.82	0.574	0.172
**ER negative (*n* = 31)**					
THBS1	1.38 ± 0.97	1.52 ± 0.81	1.42 ± 0.81	0.248	0.623
TNC	2.51 ± 0.68	2.74 ± 0.68	2.54 ± 0.78	0.145	0.855
FN	1.86 ± 0.789	2.01 ± 0.74	2.00 ± 0.80	0.31	0.571
SPARC	1.05 ± 0.86	1.55 ± 0.95	1.49 ± 0.94	0.119	0.21
α-SMA	35.7 ± 33.5	42.39 ± 28.52	47.7 ± 29.0	0.735	0.649

[a]Wilcoxon Signed Ranks Test; ER: estrogen receptor; BL: baseline; C1: cycle 1; C2: cycle 2.
